# Development of a bacteriophage-based Method for Detection of *Escherichia Coli* O157:H7 in Fresh Vegetables

**DOI:** 10.14252/foodsafetyfscj.2018010

**Published:** 2018-12-21

**Authors:** Hoang A. Hoang, Nguyen T.T. Nhung

**Affiliations:** Department of Biotechnology, Faculty of Chemical Engineering, Ho Chi Minh City University of Technology, VNU-HCM, 268 Ly Thuong Kiet, District 10, Ho Chi Minh city, Vietnam

**Keywords:** *E. coli* O157H7, bacteriophage, colorimetric detection, fresh vegetables

## Abstract

In this study, a method using a recombinant phage for detection of *E. coli* O157:H7 in fresh vegetables was investigated. Four kinds of fresh vegetables, i.e. lettuce (*Lactuca sativa*), mustard greens (*Brassica juncea*), coriander (*Coriandrum sativum*), and soybean sprouts were selected since they are commonly used in meals in Vietnam. Firstly, a phage-based method was investigated for detection of *E. coli* O157:H7 in the four types of vegetables. To support the detection by suppressing growth of background bacteria in vegetables, selective antibiotics, i.e. novobiocin (N) and vancomycin (V) in combination with BHI medium were examined. Secondly, quality of the method was evaluated in terms of sensitivity, specificity, and rapidity. The method enabled the detection of *E. coli* O157:H7 inoculated at 10^3^, 10^2^, or 10^1^ CFU/ 10 mL of sterile 0.8% NaCl containing 5 g of vegetable and in the presence of several Gram-positive and Gram-negative bacteria inoculated at 10^7^ CFU/10 mL. The time for detection was approximately 16.5 hours for *E. coli* O157:H7 inoculated at 10 CFU/10 mL of sterile 0.8% NaCl containing 5 g of vegetable. The limit of detection was considered to be 2 CFU g^-1^ vegetable.

## Introduction

Enterohemorrhagic *Escherichia coli* (EHEC) can cause severe foodborne diseases due to their Shiga toxins Sxt1 and Sxt2. Among serotypes of EHEC, *E. coli* O157:H7 is considered as the most important pathogen in relation to public health in many countries since it can causes severe bloody diarrhea and hemolytic-uremic syndrome (HUS)^1^^,^^2^^)^. *E. coli* O157:H7 can transmit to foods from the original source of animal manures^3^^)^. Therefore, since the first identification of foodborne outbreak caused by *E. coli* O157:H7 in 1982^4^^)^, it has become important to detect *E. coli* O157:H7 to prevent such outbreaks. By applying the Sorbitol-MacConkey agar plate method^5^^,^^6^^)^, low concentrations of *E. coli* O157:H7 can be detected. However, the agar plate method is time consuming since it takes more than a day for the enrichment and the formation of colonies on the agar plate. One of the approaches considered for shortening the detection time for *E. coli* O157:H7 is the use of polymerase chain reaction (PCR) for amplification of the *stx1* and *stx2* genes^7^^,^^8^^)^. Although the method is rapid, it is inadequate for distinguishing the living cells from dead cells.

The application of bacteriophages (or phages) for detection of specific bacteria is advantageous owing to the high specificity of phages in host recognition. Until now, fluorescence- or bioluminescence phage-based methods for detection of *E. coli* O157:H7 have been investigated^9^^,^^10^^)^. In those studies, the resulting fluorescence or bioluminescence could be detected using an epifluorescence microscope or a luminescence counter, respectively. Although the both methods allow selective detection of *E. coli* O157:H7 in less than one day, special apparatus are required to evaluate the results. Generally, it is easy and convenient to examine results by the colorimetric examination simply because it can be done by using a spectrophotometer that is more commonly used and easily available compared to an epifluorescence microscope or a luminescence counter. Hoang & Dien^11^^)^ constructed a recombinant phage carrying the *cytochrome c peroxidase* (*ccp*) gene encoding for the CCP enzyme and successfully applied on colorimetric detection of *E. coli* O157:H7 inoculated in a sterilized apple juice. However, condition of fresh vegetables is much different from that of sterilized apple juice. In this study, procedure of the method for detection of *E. coli* O157:H7 in fresh vegetables was investigated. In addition, quality of the method was evaluated in terms of sensitivity, specificity and rapidity.

## Materials and Methods

### Bacterial Strains and Bacteriophage

*E. coli* O157:H7 ATCC 43888 that does not produce Stx1 and Stx2 toxins was used as the host for infection of phage. Other bacterial strains were obtained from the laboratories at Department of Biotechnology, Ho Chi Minh City University of Technology. Wild-type phage PP01wt was obtained from the Yasunori Tanji lab (Tokyo Institute of Technology, Japan). It was shown to have a high host specificity^12^^)^ to *E. coli* O157:H7. Recombinant phage PP01ccp was constructed in the previous study^11^^)^.

### Vegetable Samples

Four types of store-purchased vegetables, i.e. lettuce (*Lactuca sativa*), mustard greens (*Brassica juncea*), coriander (*Coriandrum sativum*), and soybean sprouts, were purchased from a local supermarket in Ho Chi Minh City and stored at 4°C. They were washed by tap water, cut into small pieces, and weighed in 5-g portions, which were used as samples.

### Selection of Antibiotics

In order to suppress growth of background bacteria in fresh vegetables, two antibiotics, i.e. novobiocin (N) (Thermo Fisher Scientific, MA, USA) at 5 mg L^-1^ and vancomycin (V) (Thermo Fisher Scientific, MA, USA) at 10 mg L^-1^ were firstly examined for their effect on target bacteria. *E. coli* O157:H7 (E) was cultivated in BHI broth (Lab M, Lancashire, UK) containing N (BHI-E-N) or in BHI containing V (BHI-E-V) or in BHI containing N and V (BHI-E-N-V). The *E. coli* O157:H7 cultures were incubated at 37°C, 250 rpm. In addition, an *E. coli* O157:H7 culture in BHI broth without antibiotics (BHI-E) was also prepared. Optical Density at 600 nm (OD_600_) was examined every 30 minutes. In another control experiment, similar combinations were prepared with *Bacillus spp*. (B) such as BHI-B-N, BHI-B-V, BHI-B-N-V and BHI-B. The experiment was conducted in triplicate. The most suitable combination was then examined for its support for growth of *E. coli* O157:H7 in vegetables.

*E. coli* O157:H7 was artificially inoculated into fresh vegetable samples. A ten-fold dilution series was prepared to produce a range of its concentrations from 1 × 10^4^ to 1 × 10^1^ CFU per 10 mL of sterile 0.8% NaCl (Merck, Darmstadt, Germany) solution. Each of the 5-g vegetable portions was submerged in a dilution flask containing 10 mL of the *E. coli* O157:H7-inoculated solution and allowed to store overnight at 4°C. Control flasks contained each type of the vegetables in sterile 0.8% NaCl solution void of *E. coli* O157:H7 inoculum. Next day, the mixture was subjected to enrichment in 90 mL of BHI medium supplemented by N at 5 mg L^-1^ and V at 10 mg L^-1^ at 37°C with shaking 250 rpm for 15 hours. A control mixture was cultivated in 90 mL BHI medium without the antibiotics supplemented. After 15-h enrichment, the cultures were subjected to ten-fold dilution series and spread on Sorbitol-MacConkey (SMAC) agar (Himedia Laboratories, Mumbai, India). The agar plates were incubated at 37°C for 20 – 24 hours. The number of *E. coli* O157:H7 in the culture was determined from the number of colonies formed on the plates. The experiment was conducted in triplicate.

### Construction of the Phage-based Method

*E. coli* O157:H7 was inoculated into each 5-g portion of each vegetable at 1 × 10^2^, 1 × 10^1^ CFU/10-mL of sterile 0.8% NaCl as described above. After 15-h enrichment with BHI supplemented by N and V, an aliquot was drawn and subjected to centrifuge at 4°C, 6,000 rpm for 5 min. The pellet was re-suspended in BHI medium (at 1/10 of the initial volume). The number of *E. coli* O157:H7 in the medium was determined with the same method as above. Next, each prepared culture was divided into three aliquots. Two aliquots were mixed with PP01ccp or PP01wt phage lysate at multiplicity of infection (MOI) of 5.0 (phage: host). One aliquot was left blank without phage addition. All aliquots were incubated at 37°C with shaking 150 rpm for 1.5 h. They were passed through 0.45-μm filters to obtain filtrates that were then subjected to the enzyme assay. In the enzyme assay, cytochrome c from equine heart (Sigma-Aldrich, Missouri, USA) and H_2_O_2_ (Merck, Darmstadt, Germany) were used as substrates. Cytochrome c was reduced prior to the assay in accordance with the protocol described by Spinazzi et al.^13^^)^, with minor modifications. The filtrates were mixed with phosphate buffer (50 mM KH_2_PO_4_, pH 6.0), cytochrome c, and H_2_O_2_ to obtain a ten-fold dilution. The final concentrations of cytochrome c and H_2_O_2_ were 0.9 μM and 360 μM, respectively. Absorbance at 550nm (ABS_550_) of the reaction solutions was measured every minute using a spectrophotometer (CT-2200, ChromTech, Taipei, Taiwan). All the enzyme assays were conducted in triplicate.

### Specificity of the Method

Cultures of *E. coli* O157:H7 at 10^3^, 10^2^, and 10^1^ CFU/10-mL of sterile 0.8% NaCl solution were prepared as described above. A control void of an *E. coli* O157:H7 inoculation was also prepared. Different types of Gram-positive and/or Gram-negative bacteria were mixed with *E. coli* O157:H7. Gram-positive bacteria included *Bacillus cereus* ATCC 11778, *Staphylococcus aureus* ATCC 25923, *Listeria monocytogenes* ATCC 19111. Gram-negative bacteria included *Salmonella typhimurium* ATCC 14028, *Pseudomonas aeruginosa* BK. Each Gram-positive or -negative bacterial strain was cultivated in BHI until their OD_600_ of 0.1 was attained. A portion of each bacterial culture was serially diluted and spread on BHI 1.5% agar to examine the bacterial density (~10^7^ CFU mL^-1^). Another portion of each bacterial culture was centrifuged and re-suspended in sterile NaCl 0.8%. Each one mL of Gram-positive and/or -negative bacterial suspension were/was added into the total 10 mL of sterile NaCl 0.8% containing *E. coli* O157:H7 as described above.

Each of the 5-g vegetable portions was submerged in a dilution flask containing *E. coli* O157:H7 inoculated at 10^3^, 10^2^, or 10^1^ CFU/10-mL, and the Gram-positive and/or-negative bacteria inoculated at 10^7^ CFU/10-mL. All of the flasks were allowed to store overnight at 4°C. Control flasks contained each 5-g protion of the vegetable in sterile 0.8% NaCl solution and each bacterial mixture but void of *E. coli* O157:H7 inoculated. Next day, the mixtures were cultivated in 90 mL of BHI-N-V as mentioned above at 37°C, 250 rpm for 15 hours. After enrichment, the cultures were divided into two aliquots. One aliquot was subjected to ten-fold dilution series and spread on Sorbitol-MacConkey (SMAC) agar. The agar plates were incubated at 37°C for 20 – 24 hours. Concentration of *E. coli* O157:H7 in the cultures would be estimated. The other aliquot was subjected to the phage-based method using the recombinant phage PP01ccp as mentioned above. All the experiments were conducted in triplicate.

### Rapidity of the Method

*E. coli* O157:H7 at 10^4^, 10^3^, 10^2^, 10^1^ CFU/ 10-mL of sterile 0.8% NaCl solution were prepared in erlenmeyer flasks as described above. A control void of an *E. coli* O157:H7 inoculation was also prepared. Each of the 5-g vegetable portions was submerged in the flask and allowed to store overnight at 4°C. Next day, the mixture was cultivated in 90 mL of BHI-N-V medium at 37°C, 250 rpm. Two aliquots were drawn every hour. One aliquot was subjected to ten-fold dilution series and spread on Sorbitol-MacConkey (SMAC) agar. The agar plates were incubated at 37°C for 20 – 24 hours. Concentration of *E. coli* O157:H7 in the cultures would be estimated. The other aliquot was subjected to the phage-based method as mentioned above. Detection time including enrichment time of the two methods was estimated. All the experiments were conducted in triplicate.

## Results

### Selective Antibiotics

**Fig. 1.**
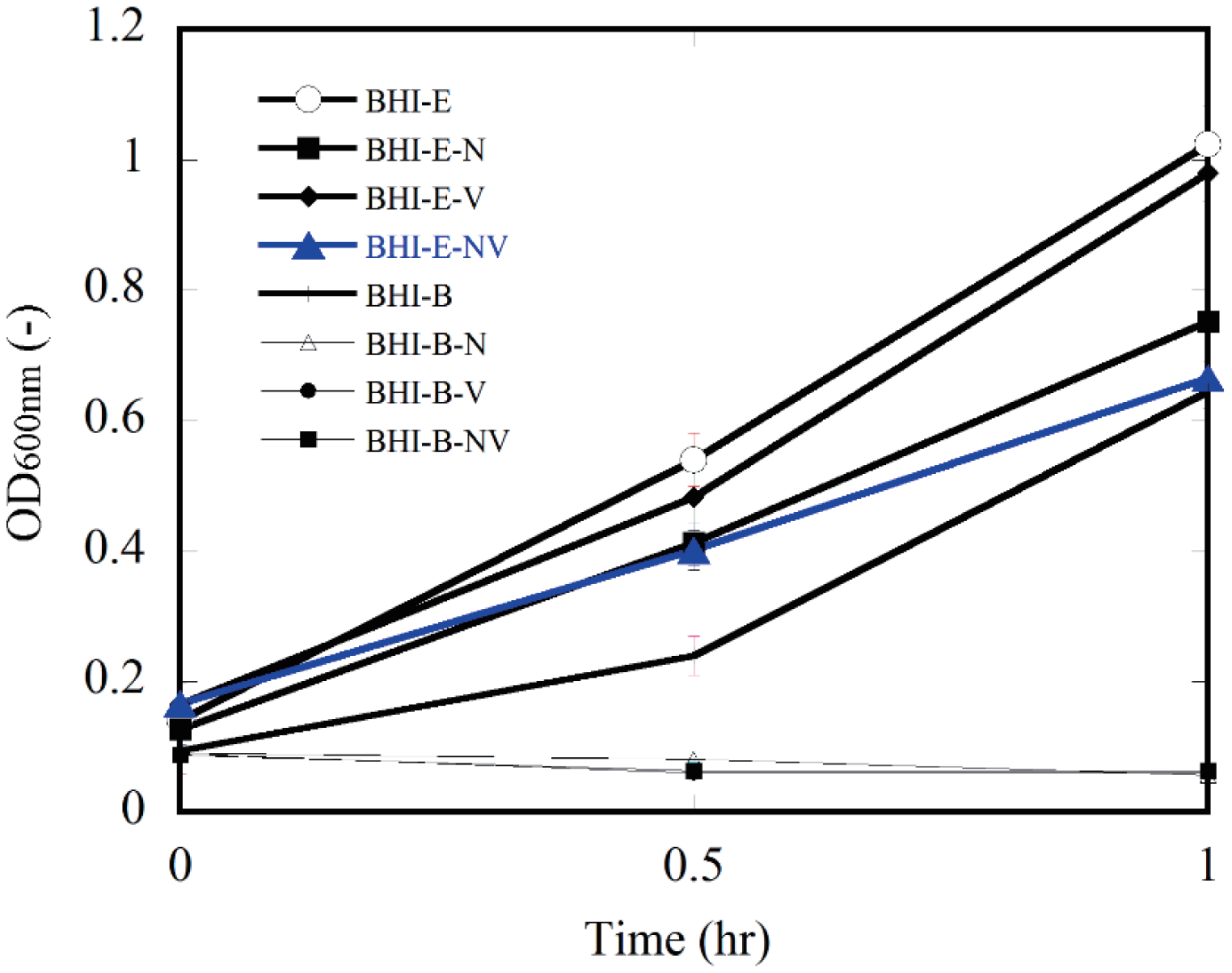
Growth of *E. coli* O157:H7 and *Bacillus spp*. in BHI supplemented by different antibiotics. Error bars indicating 95% confidence intervals for the averaged values (n = 3) are not graphically detectable at some points as the intervals are too narrow.

Antibiotics that suppress the growth of background bacteria but do not affect growth of *E. coli* can be used to support detection of *E. coli* in food samples. In this study, three antibiotic combinations in BHI medium such as BHI-N, BHI-V, and BHI-N-V were investigated. The combinations were preliminary examined their effect on the growth of *E. coli* O157:H7 and *Bacillus spp*. in medium based on OD_600_ measurement (Fig. 1). After one-hour cultivation, the OD_600_ increased sharply in cultivation of *E. coli* O157:H7. However, the OD_600_ decreased in cultivation of *Bacillus spp*. BHI medium promoted growth of *E. coli* O157:H7, while the antibiotic(s) inhibited growth of background bacteria other than *E. coli*^14^^-^^16^^)^. In order to expect the highest suppression of background bacteria in vegetables, both N and V were selected for detection of *E. coli* O157:H7 in vegetables.

*E. coli* O157:H7 cells were initially inoculated into 10 mL of sterile 0.8% NaCl containing 5-g portions of four types of vegetables. By support of the two antibiotics, *E. coli* O157:H7 from 10^4^ to 10^1^ CFU/10 mL of sterile 0.8% NaCl was totally detectable in the presence of vegetables. On SMAC agar plates, colorless colonies indicated *E. coli* O157:H7 and pink colonies indicated background bacteria. Estimated number of *E. coli* O157:H7 inoculated at 10 CFU/ 10 mL of sterile 0.8% NaCl in the presence of vegetables was shown in the Table 1. N and V involved in inhibition of background bacteria and hence supported growth of *E. coli* O157:H7. In case of *E. coli* O157:H7 inoculated without N and V supplement, *E. coli* O157:H7 was undetectable since its growth was competed by growth of background bacteria in the enrichment. No colorless colonies were found on the SMAC agar plates. In addition, in the cases of no *E. coli* O157:H7 inoculated with or without the antibiotics, there were also no colorless colonies on the SMAC agar plates. This implied *E. coli* O157:H7 did not originally exist in the vegetable samples.

**Table 1.** Log cell density of *E. coli* O157:H7 in the medium containing vegetable after the 15-h enrichment from the initial inoculation of 10 CFU/10 mL of sterile 0.8% NaCl containing 5 g of vegetable

**Sample**	Lettuce	Mustard greens	Soybean sprouts	Coriander
Vegetable + *E. coli* + antibiotics	8.77 ± 0.61	7.78 ± 0.39	7.07 ± 0.42	7.08 ± 0.51
Vegetable + *E. coli*	-	-	-	-
Vegetable + antibiotics	-	-	-	-
Vegetable	-	-	-	-

"-": undetectable

### Detection of *E. coli* O157:H7 in Vegetables Using the Phage-based Method

**Fig. 2.**
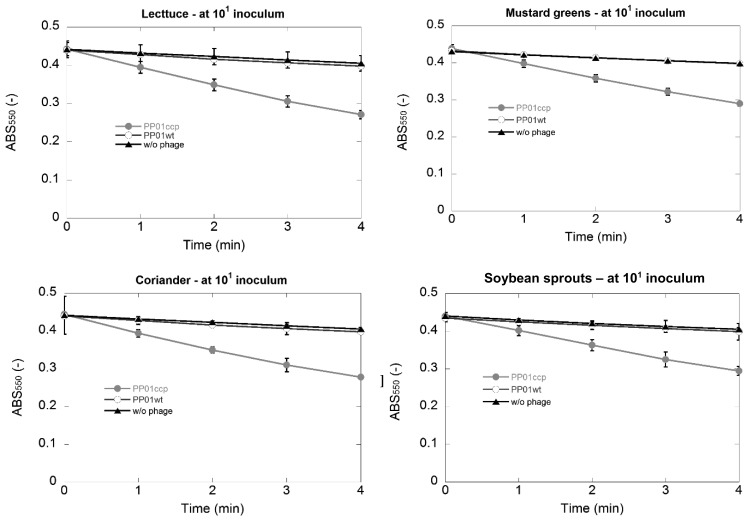
Change of absorbance at 550 nm in the detection of *E. coli* O157:H7 inoculated at 1 x 10^1^ CFU/ 10 mL of sterile 0.8% NaCl containing 5 g of vegetable. Time of the enzyme assay was shown on the horizontal axis. Error bars indicating 95% confidence intervals for the averaged values (n = 3) are not graphically detectable at some points as the intervals are too narrow.

Figure 2 shows ABS_550_ change in the detection of *E. coli* O157:H7 in four types of vegetables. A significant change in ABS_550_ appeared in the enzyme assay using the lysate obtained from infection of the recombinant phage to *E. coli* O157:H7. However, there were slight ABS_550_ changes in the assay corresponding to the control either with PP01wt or without phage addition. In other words, limit of detection (or sensitivity) of the method is 1 × 10^1^ CFU/ 10 mL of sterile 0.8% NaCl containing 5-g of vegetable.

### Specificity of the Method

**Fig. 3.**
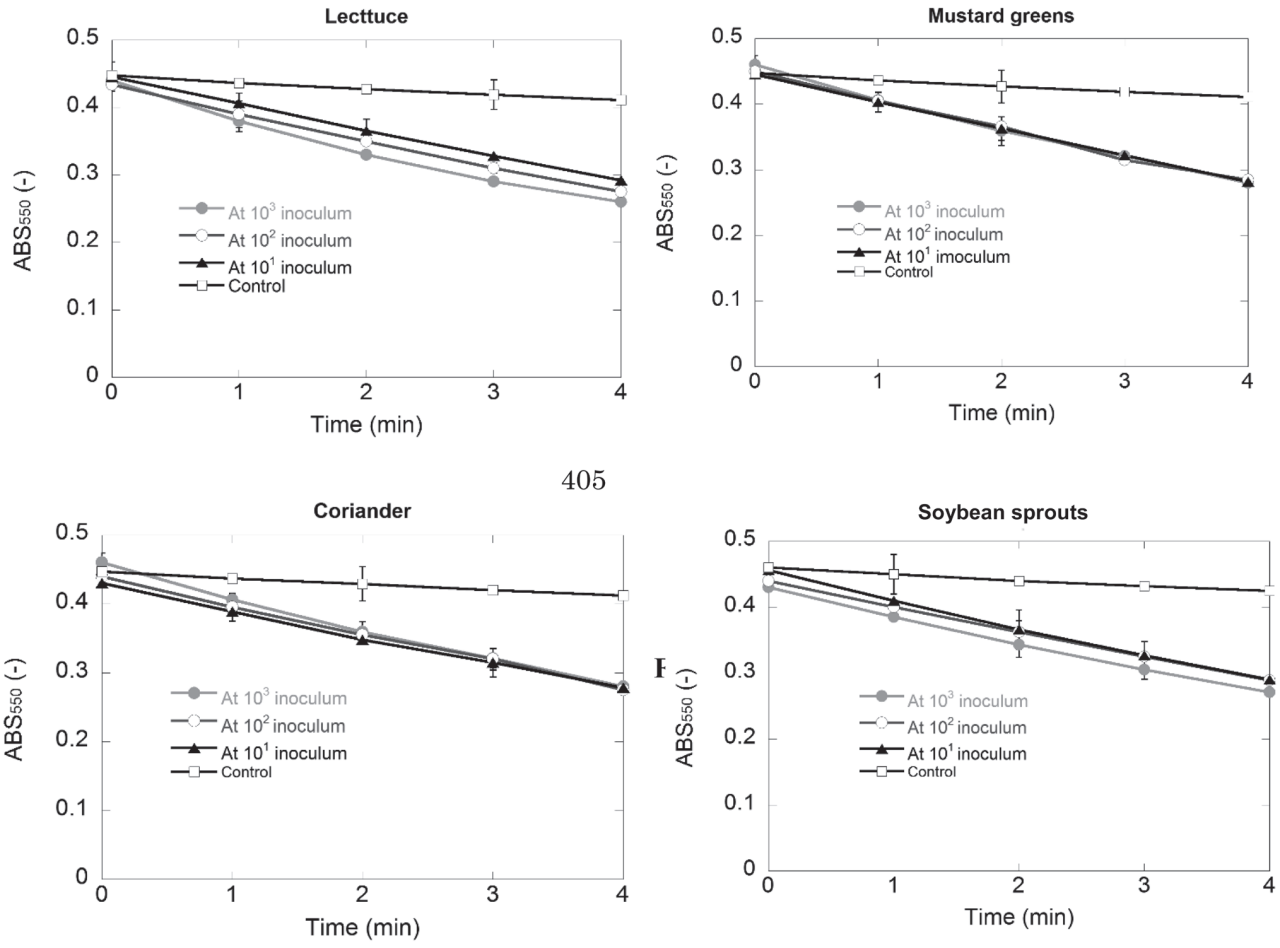
Change of absorbance at 550 nm in the detection of *E. coli* O157:H7 inoculated at 10^1^, 10^2^, or 10^3^ CFU/ 10 mL of sterile 0.8% NaCl containing 5 g of vegetable and containing Gram-positive and Gram-negative bacteria inoculated at 10^7^ CFU/10-mL. Time of the enzyme assay was shown on the horizontal axis. Error bars indicating 95% confidence intervals for the averaged values (n = 3) are not graphically detectable at some points as the intervals are too narrow.

The phage-based method was examined for its capacity in detection of *E. coli* O157:H7 in vegetables containing other Gram-positive and/or -negative bacteria. By using SMAC agar plate method, *E. coli* O157:H7 in four types of vegetable was enriched to about 10^6^ – 10^8^ CFU mL^-1^ for all cases of Gram-positive and/or -negative bacteria inoculated. Aliquots corresponding to the case of Gram-positive and Gram-negative bacteria inoculated were subjected to the phage-based method (Fig. 3). A significant change of ABS_550_ was observed in the enzyme assay derived from initial inoculation of *E. coli* O157:H7 at 10^3^ to 10^1^ CFU/ 10 mL of sterile 0.8% NaCl containing 5 g of vegetable. There was a slight ABS_550_ change in the enzyme assay corresponding to the control void of *E. coli* O157:H7 inoculated. Therefore, the phage-based method enabled the detection of *E. coli* O157:H7 in vegetables surrounded by a high concentration of different Gram-positive and Gram-negative bacteria.

### Rapidity of Method

**Fig. 4.**
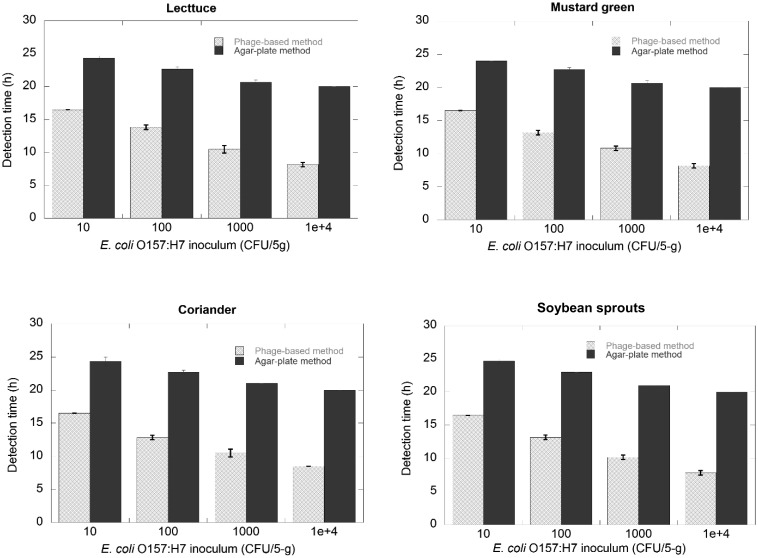
Response time profile of the detection of *E. coli* O157:H7 at different inoculums in four types of vegetables using the phage-based method and agar-plate method. Error bars indicating 95% confidence intervals for the averaged values (n = 3) are not graphically detectable at some points as the intervals are too narrow.

Figure 4 shows the time for detection of *E. coli* O157:H7 inoculated in four types of vegetables by two methods, i.e. agar plate method and phage-based method. In the agar-plate method using SMAC agar, no enrichment was required to detect *E. coli* O157:H7 inoculated at 10^4^ CFU/10 mL of sterile 0.8% NaCl containing 5 g of vegetable. Therefore, in this case, the time for detection was about 20 hours that was necessary to form colorless colonies on the agar plate. However, in the cases of *E. coli* O157:H7 inoculated at 10^3^ – 10^1^ CFU/10 mL of sterile 0.8% NaCl containing 5 g of vegetable, enrichment from one to five hours was required. The time for detection in these cases was about from 21 to 25 hours.

In the phage-based method, enrichment was required for *E. coli* O157:H7 inoculated at 10^4^ – 10^1^ CFU/ 10 mL of sterile 0.8% NaCl containing 5 g of vegetable. The time for enrichment was about 6 to 15 hours corresponding to inoculation of 10^4^ – 10^1^ CFU/10 mL of sterile 0.8% NaCl containing 5 g of vegetable. It meant that the time for enrichment of the phage-based method was longer than that of the agar-plate method. However, the time required for phage assay was only about 1.5 hours. Therefore, the time for detection of the phage-based method was about 8 to 16.5 hours. It was also shorter than the time of the agar-plate method.

## Discussion

Animal manures are considered as the original source of *E. coli* O157:H7^3^^)^. The manures are normally treated by composting to produce compost. During the composting process, *E. coli* O157:H7 is eliminated by high temperatures generated inside the composting zone^17^^)^. The composting process is usually performed by farmers especially in developing countries and may not result in mature compost that completely eliminates *E. coli* O157:H7 from the original manures. Usage of contaminated compost in agricultural practice could transmit *E. coli* O157:H7 to products, e.g. vegetables^18^^,^^19^^)^. By using fresh vegetables in meals, *E. coli* O157:H7 can infect human beings^20^^,^^21^^)^. Therefore, a sensitive, specific and rapid method for detection of *E. coli* O157:H7 has become important to prevent outbreaks of foodborne illness caused by this pathogen.

For detection of *E. coli* O157:H7 in vegetable at low concentrations such as 1 – 10 CFU/g, enrichment is always required for all of methods such as agar-plate method, DNA-based methods, biochemistry methods, phage-based method, etc. Nutrient-rich media are usually used in enrichment to enhance growth of the target bacteria at such low concentrations. However, the media also enhance growth of background bacteria that may compete to growth of the target bacteria. To overcome this problem, immunomagnetic separation (IMS) has been investigated and employed^22^^,^^23^^)^. The IMS exhibits excellent performance with respect to the rapidity and selectivity of separation. However, it is costly and requirement of additional assays is essential for completing the detection. In this study, two antibiotics, i.e. novobiocin and vancomycin were successfully selected in combination with BHI medium (BHI-N-V) to support detection of *E. coli* O157:H7 in vegetables. Novobiocin inhibits various bacteria, e.g. *Agrobacterium spp., Bacillus mycoides, Burkholderia cepacia, Staphylococcus spp.,* and *S. faecalis* but it poorly affected growth of negative bacteria^14^^)^. Vancomycin inhibits a wide range of Gram-positive and Gram-negative bacterial species, but does not affect *E. coli*^15^^,^^16^^)^. However, selection of antibiotics for detection of target bacteria in vegetables might be different from that in other environmental samples such as manure, waste water, etc. In vegetables, many bacteria exist other than *E. coli* O157:H7. Growth of these background bacteria will compete with growth of *E. coli* O157:H7 in enrichment during the detection. Due to the competition, required concentration of *E. coli* O157:H7 may not be attained to enable the detection. Usage of selective antibiotics in enrichment is much simpler and cheaper than the usage of IMS in the detection procedure of *E. coli* O157:H7 in vegetables.

Usage of BHI-N-V in the enrichment and applying the recombinant phages in the phage assay enabled detection of *E. coli* O157:H7 inoculated at 1 × 10 CFU in the 10-ml NaCl solution. Since the vegetable was added into 10-mL sterile NaCl 0.8% containing *E. coli* O157:H7 and was not taken out from the mixture during the enrichment step, it could be considered that the limit of detection of *E. coli* O157:H7 in vegetables is 1 × 10 CFU/5 g or 2 CFU/g. The limit of detection (or sensitivity) was compatible to that of the agar-plate method (the gold standard method). In addition, it is much lower than that of pre-existing phage-based methods. In research of Ripp et al^24^^)^, by overnight incubation with selective antibiotics, *E. coli* O157:H7 inoculated at 1 × 10^2^ in vegetables was detectable within 22.4 hours and no significant bioluminescence was observed from *E. coli* XL1-Blue dilutions lower than 1 × 10^2^ CFU mL^-1^.

Interference of food matrices on detection of target bacteria has shown in some other phage-based methods. The phage-based bioluminescent method^10^^)^ could not detect *E. coli* O157:H7 at less than 10^4^ CFU mL^-1^ in apple juice. The method could not detect *E. coli* O157:H7 cells at lower than 2.5 × 10^6^ CFU per 25 g ground beef^24^^)^. To overcome the interference of materials to the detection system, centrifugation or IMS is employed to separate and obtain only the target cell pellet from food matrixes^22^^,^^23^^)^. In this study, centrifugation was also applied to minimize possible effect of vegetables on the phage assay. It could then enable detection of *E. coli* O157:H7 in the four kinds of vegetables as low as 2 CFU g^−1^ within 16.5 hours (including 15 hours for enrichment and about 1.5 hours for the phage assay).

The method has a high specificity since it enables the detection of *E. coli* O157:H7 in vegetables surrounded by a high concentration of different Gram-positive and Gram-negative bacteria. In addition, it was also demonstrated PP01 phage was highly specific to *E. coli* O157:H7^12^^)^. Therefore, false-positive detection in vegetables that may be contaminated with other *E. coli* strains could be avoided. The method developed in this study can be considered as the first successfully performed phage-based colorimetric detection of *E. coli* O157:H7 in vegetables. In future studies, the method will be examined for the detection of *E. coli* O157:H7 in meat samples.
